# Impact of Body Adiposity (Lean and Fat Mass Distribution) on Bone Mineral Density in Postmenopausal Indian Women: A Cross-Sectional Study

**DOI:** 10.7759/cureus.85745

**Published:** 2025-06-11

**Authors:** Agrata Sharma, Piyush Pathak, Rajinder Tonk, Reema Malik, Amit Raj

**Affiliations:** 1 Department of Neurology, All India Institute of Medical Sciences, Bhopal, Bhopal, IND; 2 Department of Gastroenterology, All India Institute of Medical Sciences, Bhopal, Bhopal, IND; 3 Department of Medicine, Atal Bihari Vajpayee Institute of Medical Sciences and Dr. Ram Manohar Lohia Hospital, New Delhi, IND; 4 Department of Conservative Dentistry and Endodontics, Santosh Dental College, Ghaziabad, IND; 5 Department of Prosthodontics, All India Institute of Medical Sciences, New Delhi, New Delhi, IND

**Keywords:** body composition, bone mineral density, obesity, osteoporosis, postmenopausal women

## Abstract

Background

Osteoporosis and obesity are prevalent health conditions that share overlapping risk factors and physiological consequences. After menopause, hormonal changes impact both bone strength and fat distribution. Although fat mass (FM) and lean mass (LM) are important components of body composition, their separate effects on bone mineral density (BMD) are not well understood, especially among Indians.

Objective

The main objective of this study is to assess how body adiposity, such as FM and LM, is related to BMD in postmenopausal Indian women.

Methods

A total of 36 postmenopausal women participated in a cross-sectional study at a tertiary care hospital. Participants underwent dual-energy X-ray absorptiometry (DEXA) scans (Hologic Inc., Marlborough, MA, USA) to measure BMD at the spine, hip, wrist, and whole body, along with body composition assessments. Women with secondary causes of osteoporosis, or those on medications affecting bone metabolism, were excluded. Correlations between FM, LM, and BMD were assessed using statistical analysis.

Results

Among the participants (n = 36), 17 (47.2%) had osteopenia, nine (25.0%) had osteoporosis, and 10 (27.8%) had normal BMD. FM showed significant positive correlations with hip BMD (r = 0.457, p = 0.005), lumbar spine BMD (r = 0.373, p = 0.025), total body BMD (r = 0.349, p = 0.037), and whole-body bone mineral content (WB-BMC) (r = 0.498, p = 0.002). After statistical adjustment, LM correlations were weaker and not statistically significant.

Conclusion

In Indian postmenopausal women, FM is a stronger predictor of BMD than LM. These findings underscore the importance of considering body fat when assessing and managing osteoporosis risk in this population.

## Introduction

Osteoporosis and obesity are two common community health issues with significant impact on morbidity and mortality, and their numbers have been increasing over the years. Initially, it was considered that these two disorders were unrelated to each other, but with increasing research, it has been found that these two disorders share several common features. Both clinically and at the molecular level, there are several shared characteristics and common environmental factors. Osteoporosis is defined as microstructural degradation of bone tissue and is measured as bone mineral density (BMD) [1]. BMD is estimated by T-scores and Z-scores. The T-score, i.e., 2.5 standard deviations beneath the reference mean established for gender- and age-matched healthy controls (-2.5), is defined as osteoporosis. Postmenopausal females with a T-score less than -1.0 are also at risk of osteoporosis. The Z-score is also used to assess osteoporosis risk and is expressed as a standard score, indicating how far a measurement differs from age-matched BMD norms. Low BMD is delineated by a Z-score under -2.0. One standard deviation decrease in BMD leads to a two- to three-fold increase in fracture risk. Osteopenia can be defined as having a T-score between -2.0 and -1.0. In postmenopausal females, 50% of fractures occur in the osteopenia group. Dual-energy X-ray absorptiometry (DEXA) is generally utilized for evaluating BMD and is regarded as the gold standard for measurement [2]. DEXA assesses BMD by measuring bone mineral content (BMC) of the entire body, as well as specific regions such as the distal radius, proximal femur, and lumbar spine.

Various factors affect BMD, including environmental and genetic influences. Among these, body weight is the most vital influencing factor [3-6]. Previous literature shows both positive and negative relationships between BMD and body weight [7-11]. Body mass index (BMI) is a simple and widely applied metric for classifying weight status as underweight, normal weight, overweight, or obese. It is calculated as weight in kilograms divided by height in meters squared (kg/m²). A BMI <18.5 kg/m² is classified as underweight, 18.5-22.9 kg/m² as normal weight, 23-24.9 kg/m² as overweight, and ≥25 kg/m² as obese. While widely used, BMI has limitations, as it does not distinguish between lean mass (LM) and fat mass (FM), nor does it assess regional fat distribution, such as abdominal adiposity.

There are several biological links between adipose tissue and bone tissue, given their shared progenitor cells [12]. Increased FM in overweight and obese individuals results in greater mechanical load and stress on the bones, which can enhance BMD. Beyond mechanical factors, fat cells (adipocytes) contribute endocrinologically by producing estrogen, which suppresses bone resorption by inhibiting osteoclasts and promotes bone formation by stimulating osteoblasts [7,13]. Total body weight comprises FM, LM, and skeletal/connective tissue mass.

Approximately 95% of total body weight is composed of FM and LM. Research exploring associations between FM, LM, and BMD has yielded inconsistent findings. Menopause alters body composition, leading to increased total and central (android) adiposity and reduced peripheral (gynoid) fat. These changes are often accompanied by declines in both regional and whole-body BMD (WB-BMD) [14]. Few studies have specifically evaluated the relationship between BMD and fat distribution among Indian women [15]. Therefore, our single-center, cross-sectional study aimed to investigate how body adiposity, specifically FM and LM, affects BMD in postmenopausal Indian women.

## Materials and methods

This investigation was executed at a tertiary health care center in northern India, after obtaining permission from the Institutional Human Ethics Committee (approval no. 1-40128/2014/1WThesis/PGIMER-RMLH). Written informed consent was obtained from all participants before registration.

Study population

Consecutive postmenopausal women attending the medicine OPD between November 2014 and March 2016 were requested to participate in the study. The permanent cessation of menstruation for 12 months is known as menopause, which is diagnosed retrospectively by taking a history of amenorrhea. It is due to the loss of ovarian follicular function. Patients unwilling to participate or having chronic kidney disease, chronic liver disease, malignancy, rheumatological disorders, endocrine disorders, vitamin D deficiency, or those on medications that interfere with bone metabolism - such as oral glucocorticoids, immunosuppressants (cyclosporine, tacrolimus, and methotrexate), anti-convulsants (phenytoin, carbamazepine, and phenobarbitone), aromatase inhibitors, bisphosphonates, and lithium - assessed based on history and previous records, were excluded. Also, those who were non-ambulatory, chronic alcoholics, chronic smokers, post-hysterectomy, and oophorectomy patients were excluded.

Smoking is categorized as non-smokers and smokers. Drinking is characterized by drinkers and non-drinkers. Drinkers are those who have consumed any alcoholic beverage more than once in the past month. Non-drinkers are those who have not consumed any alcoholic beverages during the last month.

Procedure

Complete sociodemographic information, including age, gender, occupation, educational attainment, and comorbidities, was documented after a comprehensive physical examination to rule out any other systemic disease or related systemic issue. Parathyroid hormone (PTH), 25-hydroxy vitamin D, and serum calcium levels within the normal range were included in this study after laboratory tests were performed. Three BMD assessments were performed using a whole-body DEXA scan (Hologic Inc., Marlborough, MA, USA) at the right hand, the lumbar spine, and the sub-regions of the femur. The following World Health Organization criteria were applied for classifying patients whose BMD was determined by DEXA: osteoporosis: “BMD -2.5 and below; severe osteoporosis: T score -2.5 and below with delicacy fractures; low bone density (osteopenia): T score between -1.0 and -2.5; and normal BMD: T score -1.0 or above.” Using the same DEXA instrument, body composition measures (FM, LM, android FM, and gynoid FM) were recorded. A manufacturer-supported software tool was used to define the android and gynoid zones.

## Results

There were 36 postmenopausal women in the study, and their ages ranged from 39 to 85 years. The mean age was 55.11 ± 11.2 years. The largest group of women (n = 14, 38.39%) was aged between 51 and 60. The height of the subjects ranged from 140 to 163 cm, with an average of 152.47 ± 5.65 cm. Their weight ranged from 35 to 165 kg, with an average of 59.86 ± 22.05 kg. The BMI values were between 15.76 and 65.27 kg/m², and the average was 25.67 ± 8.7 kg/m². Table 1 shows that the mean WB-BMD was 0.99 ± 0.11 g/cm², and WB-BMC was 1572.95 ± 282.79 g. The mean T-score was -1.55 ± 1.18. Regional BMD values included the hip BMD (H-BMD) (0.83 ± 0.14 g/cm²), lumbar spine BMD (L-BMD) (0.75 ± 0.14 g/cm²), and wrist BMD (W-BMD) (0.43 ± 0.08 g/cm²). FM averaged 21,467.86 ± 8417.89 g, LM averaged 32,222.08 ± 6134.18 g, with android and gynoid FMs at 1672.06 ± 800.15 g and 3135.53 ± 1078.16 g, respectively.

**Table 1:** Bone density and adiposity parameters of the study group This table provides the descriptive statistics of the study population, including BMD, BMC, and adiposity measures. WB-BMD, Whole-body bone mineral density; H-BMD, Hip bone mineral density; L-BMD, Lumbar spine bone mineral density; W-BMD, Wrist bone mineral density; WB-BMC, Whole-body bone mineral content

Parameters	Mean ± SD	Median	Min-Max	Interquartile Range
WB-BMD (g/cm^2^)	0.99 ± 0.11	0.998	0.8 - 1.17	0.901 - 1.091
WB-BMC (g)	1572.95 ± 282.79	1518.67	1105.89 - 2136	1339.7 - 1797.855
WB-T Score	-1.55 ± 1.18	-1.45	-3.5 to 0.8	-2.5 to -0.950
H-BMD (g/cm^2^)	0.83 ± 0.14	0.82	0.65 - 1.12	0.707 - 0.956
L-BMD (g/cm^2^)	0.75 ± 0.14	0.73	0.53 - 1.18	0.669 - 0.815
W-BMD (g/cm^2^)	0.43 ± 0.08	0.44	0.21 - 0.59	0.366 - 0.472
Fat Mass (g)	21467.86 ± 8417.89	20322	7864.3 - 40389	15724.5 - 28738.050
Lean Mass (g)	32222.08 ± 6134.18	30278.9	24729.7 - 49407.2	26922.650 - 37444
Android Fat Mass (g)	1672.06 ± 800.15	1662.5	359 - 3342	1095 - 2367
Gynoid Fat Mass (g)	3135.53 ± 1078.16	2985.5	1160 - 5484	2232 - 3975

Table 2 shows that, after adjusting for age and weight, FM showed significant positive correlations with WB-BMD (r = 0.372, p = 0.030), H-BMD (r = 0.417, p = 0.014), L-BMD (r = 0.377, p = 0.028), and WB-BMC (r = 0.473, p = 0.005). In contrast, LM exhibited weaker and statistically non-significant correlations with all bone parameters, including WB-BMD (r = 0.261, p = 0.136) and WB-BMC (r = 0.328, p = 0.058).

**Table 2:** Correlation between FM and LM with BMD and BMC after age and weight adjustment This table, after adjusting for age and weight, shows that fat mass has significant positive correlations with WB-BMD (r = 0.372, p = 0.030), H-BMD (r = 0.417, p = 0.014), L-BMD (r = 0.377, p = 0.028), and WB-BMC (r = 0.473, p = 0.005). WB-BMD, Whole-body bone mineral density; H-BMD, Hip bone mineral density; L-BMD, Lumbar spine bone mineral density; W-BMD, Wrist bone mineral density; WB-BMC, Whole-body bone mineral content

Parameters	Age and Weight Adjusted			
	Fat Mass		Lean Mass	
	Correlation Coefficient	p-value	Correlation Coefficient	p-value
WB-BMD	0.372	0.030	0.261	0.136
H-BMD	0.417	0.014	0.212	0.230
L-BMD	0.377	0.028	0.152	0.391
W-BMD	0.247	0.159	0.174	0.326
WB-BMC	0.473	0.005	0.328	0.058

After age and weight adjustment, FM was directly associated with WB-BMC (p = 0.005), WB-BMD (p = 0.372), H-BMD (p = 0.014), and L-BMD (p = 0.028). There was no statistical connection between LM and WB bone density, or at any other point. Table 3 depicts that, after adjusting for age and FM, LM was significantly associated with WB-BMC (r = 0.380, p = 0.027), but not with WB-BMD (r = 0.022, p = 0.900). FM showed a positive but non-significant correlation with WB-BMD (r = 0.276, p = 0.114). Among anthropometric parameters, height showed a strong positive correlation with WB-BMC (r = 0.497, p = 0.002), whereas BMI and weight had no meaningful associations with BMD or BMC.

**Table 3:** Correlation between adiposity mass (lean and fat), anthropometric parameters, and BMD and BMC after adjustments This table, after adjusting for age and FM, shows that LM has a significant positive correlation with WB-BMC (r = 0.380, p = 0.027), while its association with WB-BMD remained non-significant. WB-BMC, Whole-body bone mineral content; WB-BMD, Whole-body bone mineral density; H-BMD, Hip bone mineral density; L-BMD, Lumbar spine bone mineral density; W-BMD, Wrist bone mineral density; BMI, Body mass index; LM, Lean mass; FM, Fat mass

Parameters	Age and FM Adjustment		Age and LM Adjustment		Age Adjustment					
	LM		FM		Weight		Height		BMI	
	Correlation Coefficient	p-value	Correlation Coefficient	p-value	Correlation Coefficient	p-value	Correlation Coefficient	p-value	Correlation Coefficient	p-value
WB-BMD	0.022	0.900	0.276	0.114	0.0004	0.998	0.089	0.610	-0.0003	0.998
H-BMD	0.039	0.827	0.370	0.031	0.194	0.265	0.230	0.184	0.157	0.369
L-BMD	-0.070	0.695	0.342	0.048	0.048	0.784	0.050	0.774	0.047	0.788
W-BMD	0.016	0.928	0.181	0.305	0.003	0.986	0.207	0.232	-0.026	0.880
WB-BMC	0.118	0.505	0.380	0.027	0.175	0.316	0.497	0.002	0.093	0.595

No statistically important association was found between total and regional BMD after adjusting for FM and LM. FM was interrelated with total BMD (p = 0.276), as well as with regional BMD at the lumbar (p = 0.048) and hip regions (p = 0.031) after FM adjustment. Only height was significantly correlated with WB-BMC (p = 0.002) among anthropometric parameters. Table 4 shows that increasing age was generally associated with a decline in body composition and bone parameters. The strongest inverse correlation was observed between age and WB-BMC (r = -0.317, p = 0.059), followed by LM (r = -0.275, p = 0.105). FM (r = -0.092, p = 0.592), WB-BMD (r = -0.156, p = 0.363), and the WB T-score (r = -0.232, p = 0.173) also showed negative trends, though not statistically significant. Android FM had a negligible positive correlation (r = 0.010, p = 0.956), while gynoid FM showed a weak negative correlation (r = -0.139, p = 0.417).

**Table 4:** Correlation between age and various parameters This table presents the relationship between age and various body composition and bone parameters, showing a general trend of decline with increasing age. The strongest inverse correlation was noted between age and WB-BMC (r = -0.317, p = 0.059), followed by lean mass (r = -0.275, p = 0.105). WB-BMC, Whole-body bone mineral content; WB-BMD, Whole-body bone mineral density

Parameters	Regression Coefficient (B)	Std. Error	Correlation Coefficient	p-value
Age and Fat Mass (g)	-69.341	128.125	-0.092	0.592
Age and Lean Mass (g)	-150.260	90.157	-0.275	0.105
Age and Android Fat Mass (g)	0.687	12.230	0.010	0.956
Age and Gynoid Fat Mass (g)	-12.547	15.285	-0.139	0.417
Age and WB-BMC (g)	-8.113	4.157	-0.317	0.059
Age and WB-BMD (g/cm²)	-0.002	0.002	-0.156	0.363
Age and WB-T Score	-0.025	0.018	-0.232	0.173

There was no substantial relation between age and other parameters, but with age, BMD and BMC decrease, and fat parameters also decrease - except for android FM, which showed a slightly positive, although not significant, correlation. A decrease in FM was far less (B = 69.341) compared to the decrease in LM with age (B = -150.260). Table 5 demonstrates that FM was significantly correlated with WB-BMC (r = 0.498, p = 0.002), WB-BMD (r = 0.349, p = 0.037), H-BMD, and L-BMD. LM showed significant associations with WB-BMC (r = 0.418, p = 0.011) and FM (r = 0.562, p = 0.0004), but not with WB-BMD. Regional fat distribution - especially gynoid and android fat - was also positively linked with hip and lumbar spine BMD. WB-BMD was strongly associated with regional BMDs across the hip, lumbar spine, and wrist.

**Table 5:** Simple correlation between various bone and adiposity parameters This table illustrates multiple significant correlations between body composition parameters and BMD. * denotes p < 0.05; ** denotes p < 0.01. WB-BMD, Whole-body bone mineral density; H-BMD, Hip bone mineral density; L-BMD, Lumbar spine bone mineral density; W-BMD, Wrist bone mineral density; WB-BMC, Whole-body bone mineral content

Parameters	Correlation Coefficient	p-value
Fat Mass and WB-BMC	0.498	0.002
Fat Mass and WB-BMD	0.349	0.037
Fat Mass and H-BMD	0.457	0.005
Fat Mass and L-BMD	0.373	0.025
Fat Mass and W-BMD	0.24	0.158
Lean Mass and Fat Mass	0.562	0.0004
Lean Mass and WB-BMC	0.418	0.011
Lean Mass and WB-BMD	0.241	0.158
Android Fat and H-BMD	0.360	0.031
Android Fat and L-BMD	0.296	0.080
Android Fat and W-BMD	0.099	0.567
Android Fat and WB-BMD	0.282	0.095
Gynoid Fat Mass and H-BMD	0.486**	0.003
Gynoid Fat Mass and L-BMD	0.400*	0.016
Gynoid Fat Mass and W-BMD	0.270	0.111
Gynoid Fat Mass and WB-BMD	0.298	0.077
WB-BMD and H-BMD	0.597	0.0001
WB-BMD and L-BMD	0.651	0.00002
WB-BMD and W-BMD	0.496	0.0021

With the increase in FM by 266.51 units, BMD increases by 0.01 units. With the increase in FM by 14.62 units, BMC increases by 1 unit. There was no significant relation between LM and BMD or BMC. Android FM was statistically linked to H-BMD (p = 0.031, r = 0.360). Gynoid FM presented a substantial and positive relationship with H-BMD (r = 0.486) and L-BMD (r = 0.400). There was a clear link discovered between gynoid FM and H-BMD (p = 0.003, r = 0.486). Total body BMD was notably and positively correlated to regional BMD, maximum at L-BMD (r = 0.651), followed by H-BMD (r = 0.597) and W-BMD (r = 0.496). WB-BMD was positively interrelated with L-BMD (r = 0.651).

## Discussion

Menopause leads to changes in body adiposity parameters and distribution, along with accelerated bone loss. There is also a documented increased prevalence of bony fractures in the postmenopausal group, which substantially impacts health, with increased health expenditure. Osteoporotic fractures, especially Colles, proximal femur, and vertebrae fractures, are associated with excess mortality. Therefore, we investigated how different levels of body fat affect BMD. Osteoporosis is more common among people living in Asia than in Western countries. In India, more than 61 million people have osteoporosis, and it is more common in women [16,17]. Indian women had 1.5 standard deviations less BMD than women in the Western population [18]. A survey from Chandigarh found that almost 53% of people had osteoporosis or osteopenia [19]. As part of this investigation, 47.22% of the study group had osteopenia, and 25.00% had osteoporosis. The anthropometric characteristics of the study group were comparable to those reported in previous research, as shown in Table 6.

**Table 6:** Comparative anthropometric characteristics of postmenopausal women across different studies This table summarizes the subject characteristics in comparison to previous studies, showing that the present study's values are comparable to those reported earlier. BMI, Body mass index

Age (year)	Height (cm)	Weight (kg)	BMI (kg/m²)	References
58.1 ± 5.0	162.0 ± 5.00	65 ± 9.4	24.6 ± 3.5	[20]
57.9 ± 4.0	163.9 ± 6.7	67.4 ± 11.6	25.1 ± 4.2	[18]
64.2 ± 6.5	160.5 ± 5.9	65.6 ± 11.0	25.5 ± 4.2	[2]
62.2 ± 1.7	165 ± 2.3	65.3 ± 3.1	23.98 ± 4.2	[21]
63.0 ± 7	157 ± 0.06	64.6 ± 9.3	26.2 ± 3.6	[19]
63.1 ± 9.5	150.7 ± 6.0	54.2 ± 9.3	23.8 ± 3.8	[10]
60 ± 3.7	161.9 ± 6.3	67.6 ± 14.3	26.1 ± 6.0	[22]
55.1 ± 11.2	152 ± 5.65	59.86 ± 22.05	25.67 ± 8.7	Present study

Correlation of various bone and body composition parameters with age

Our study confirms a postmenopausal reduction in total body bone mass, consistent with earlier findings [27-29]. Total BMC and BMD showed a progressive decline with age: for BMC, r = -0.317; p = 0.059, and for BMD, r = -0.156; p = 0.363 - although this was not significant in our study. These BMD values are also consistent with the range reported in previous studies, as stated in Table 7. No significant change in FM and LM was documented with age, although a progressive decline in LM (r = -0.275) was more marked than in FM (r = -0.092). This pattern of body composition change is consistent with other studies (Table 8). Among FM parameters, android mass showed a positive correlation (r = 0.10), and gynoid mass (r = -0.139) showed a negative correlation with age, although neither was significant. This change in body composition was similar to that seen in other studies, except for a significant rise in FM and weight with age observed in some, which might be explained by the poor nutritional status of our study group [30,31].

**Table 7:** Comparison of BMD parameters This table summarizes the BMD parameters across studies, showing that the BMD values found in this study are largely within the range documented in other studies. WB-BMD, Whole-body bone mineral density; H-BMD, Hip bone mineral density; L-BMD, Lumbar spine bone mineral density

WB-BMD (g/cm²)	L-BMD (g/cm²)	H-BMD (g/cm²)	References
1.06 ± 0.09	1.03 ± 0.14	0.85 ± 0.10	[23]
1.089 ± 0.088	1.085 ± 0.166	0.869 ± 0.109	[19]
1.027 ± 0.091	0.976 ± 0.160	0.810 ± 0.112	[11]
1.148 ± 0.011	1.152 ± 0.091	0.962 ± 0.015	[5]
1.082 ± 0.08	1.055 ± 0.166	0.844 ± 0.015	[24]
1.031 ± 0.106	0.812 ± 0.142	0.612 ± 0.117	[13]
0.99 ± 0.41	0.99 ± 0.72	0.99 ± 0.89	[10]
0.099 ± 0.11	0.75 ± 0.14	0.83 ± 0.14	Present study

**Table 8:** Comparison of adiposity parameters, including total body FM and LM This table presents the adiposity parameters across various studies, showing that the fat mass and lean mass values observed in the present study align closely with previously reported data. FM, Fat mass; LM, Lean mass

Total body FM (kg)	Total body LM (kg)	References
24.9 ± 7.7	37.2 ± 3.6	[20]
23.8 ± 8.5	40.5 ± 4.6	[16]
26.5 ± 5.4	37.6 ± 4.6	[25]
24.5 ± 5.8	32.7 ± 5.8	[12]
36.0 ± 5.8	38.3 ± 4.0	[17]
20.4 ± 5.6	34.2 ± 4.6	[26]
26.9 ± 8.7	36.2 ± 4.8	[3]
21.46 ± 8.4	32.22 ± 6.14	Present study

Correlation between WB-BMD and BMD at other points

Our study also showed a very strong correlation between total body BMD and BMD at various sites, the strongest being between WB-BMD and H-BMD (r = 0.651; p = 0.0001), followed by WB-BMD and L-BMD (r = 0.591; p = 0.00002), and WB-BMD and W-BMD (r = 0.491; p = 0.0021). This correlation is important, as hip and spinal fractures rise exponentially with age, while only a modest age-related increase in Colles fractures has been observed in studies [32].

Correlation between body composition and bone mass parameters

In agreement with findings from other studies, we observed that FM was a more important predictor of BMD of the total body (r = 0.349, p = 0.037), hip (r = 0.457, p = 0.005), and lumbar spine (r = 0.373, p = 0.025), than LM. The strongest correlation was found between FM and WB-BMC (r = 0.498, p = 0.002) [33].

Our study revealed a positive relationship between LM and WB-BMC (r = 0.418)/WB-BMD (r = 0.241), although it was only significant, after age adjustment, with WB-BMC (p = 0.011). This was in contrast to other studies that identified LM as a major predictor of BMD [34]. There are a large number of factors that explain the association between bone and adiposity (FM and LM), including the mechanical stress of increasing adiposity and the conversion of adrenal androstenedione to oestrone in FM. A negative impact of FM on BMD in premenopausal women has been reported [34].

Inflammatory cytokines produced by FM may negatively affect bone density. At the same time, estrogen produced by the FM may positively affect bone mass. Negative affect may be counterbalanced by positive affect and may increase BMD, as shown in our study. Lean body mass may also similarly affect the BMD [35]. If this were the actual mechanism, then LM could also be connected to BMD, and weight would be more closely related to BMD than FM. In our study, fat correlated more with BMD than weight. Also, height was more significantly correlated with BMD than weight. Another study showed that the fat-BMD relationship is not dependent on serum estrone levels [36]. This suggests that other mechanisms are also involved, such as the endocrinology of adiposity. Sex hormone-binding globulin is decreased with increasing adiposity, which leads to elevated levels of estrogen, insulin, and leptin. These three hormones are known to stimulate osteoblasts and, hence, result in increased BMD. Two regulators of carbohydrate metabolism, i.e., amylin and CGRP (calcitonin gene-related peptide), also influence bone health. Both these peptides produce insulin resistance in bone [37]. Thus, this results in a reduction in total body fat by shifting glucose from muscle cells to adipocytes. They also inhibit bone resorption and would increase BMD. These are certain possible mechanisms, but they are not yet proven or certain. Further research needs to be done targeting the bone-fat relationship.

Correlation between fat distribution and BMD

This study observed a positive association between android FM (r = 0.282) and gynoid FM (r = 0.298) mass with BMD. One study reported a negative correlation between android FM and BMD, while another found that android fat was positively associated with BMD in males, and gynoid FM was negatively associated with BMD in females [38,39]. To our knowledge, this study is the first in Indian postmenopausal females to establish the connection between FM and BMD. Several notable findings were observed: first, it was a single-institution study, which may have ensured internal validity; second, the population was clearly defined with respect to vitamin levels.

However, this study has certain limitations that should be considered when interpreting the findings. The sample size was relatively small and drawn from a single center, which may limit the generalizability of the results. Additionally, the cross-sectional design prevents causal inferences. Factors such as physical activity levels, dietary intake, and hormonal status, which may influence BMD, were not assessed in this study.

## Conclusions

This research analyzed the influence of body adiposity on BMD in healthy 36 postmenopausal women after excluding secondary causes of osteoporosis. With age, BMD showed a decreasing trend (r = -0.156); among fat parameters, LM (r = 0.275) was decreasing at a faster rate than FM (r = 0.092). A statistically significant connection was found between WB-BMD and regional BMD: WB-BMD and H-BMD (r = 0.597, p = 0.0001); WB-BMD and L-BMD (r = 0.651, p = 0.00002); and WB-BMD and W-BMD (r = 0.496, p = 0.0021). There was also a significant statistical relation between FM and WB-BMD (p = 0.037), as well as between FM and WB-BMC (p = 0.498). FM was significantly linked with regional BMD at H-BMD (p = 0.005) and L-BMD (p = 0.025), but not at W-BMD (p = 0.158). FM and LM showed a significant correlation (p = 0.0004). LM was favorably linked with WB-BMD (r = 0.241) and WB-BMC (r = 0.418), although the relationship was statistically insignificant. There was no substantial correlation between FM distribution (android and gynoid FM) and WB-BMD or WB-BMC. The study concluded that total body FM is the most important analyzer among adiposity parameters of BMD in the postmenopausal population.
